# The Rheumatic Element in Chorea of Early Childhood

**Published:** 1887-08

**Authors:** 


### THE RHEUMATIC ELEMENT IN CHOREA OF EARLY CHILDHOOD.

DR. Octavius Sturges, of London, says: Chorea and rheumatic arthritis, whether in union or apart, are first discernable at a period of life which though past infancy is yet near enough to that age to retain some of its characteristics. Now, in infancy morbid phenomena are the most generalized, and concern mainly the nervous system, the excitability of whose reflex centers is excessive. At this age impressions of the most various kinds, whether central or peripheral, have a common issue in convulsion—convulsion which if not general is so variously and capriciously bestowed that the part selected for spasm gives but little guide to the source of it. Even when infancy has passed, and convulsion begins to limit its area, there is still a remarkable indifference and changefulness in its modes of display. Thus, with a young child spasm of the larynx will very readily pass to the diaphragm, so that there shall be stridor or laryngismus one day or one hour, and dyspnœa the next. And speaking generally, many central lesions—cerebral hæmorrhage, tumor, embolism—which in after life give definite notice of their seat by means of the particular muscles they affect, are in early childhood so confused together that any attempt at localization would be futile. The nervous function is as yet imperfectly differentiated; it is unable to appreciate various sources and modes of irritation, or to separate them one from the other. And so long as this process of differentiation remains incomplete we should expect (as we find) that those morbid phenomena which, as there is independent reason for believing, have a real kindred—depending, so to say, upon the stimulation of neighboring centers — should for the while be merged and confused together. But growth and use have a constant tendency to draw them apart. Differentiation becomes more accurate, while at the same time external and accidental circumstances impart their own bias in this direction or in that.

Let me make my meaning plain by an illustration. Take any of the examples that have been already quoted of young children exhibiting side by side chorea and rheumatic arthritis. In these we have a group of symptoms, motor, cardiac, articular, signals in common of some nervous disturbance. Here, no doubt, are the elements out of which in later life we construct two diseases. But at present there are not two, but one. And by their appearance at this exceptionally early period of life they prove a special liability on the part of their subject both to chorea and to rheumatic arthritis. Now, there are in the world choreic inducements, and rheumatic inducements, and there are epochs of life which are favorable now to the development of the one disease, now to the other. And so in life's progress it will depend very much upon accident whether the child supposed shall develop chorea or rheumatism. She is ready for both, yet with a varying preference which is determined mainly by age. But this comparative indifference is of short duration. Let a child be encountered with consequent rheumatism, or a sudden alarm with consequent chorea, and either of these disorders will impress its own likeness and impart a proneness to repetition. But it is not the cause alone that has to be considered, there is the season of life as well. Chorea, for reasons that need not now be discussed, is a child's liability, favoring the female sex. Rheumatism is a young adult liability, with no marked sex preference except (as has been shown) in early childhood. We see, therefore, in that later period of childhood, when the two diseases have, so to speak, joint reign, the one or the other occurring by preference according as accident has determined the character of the early attacks, while still the liability to suffer in either way is not yet at one end. Up to a certain age fright or nervous excitement may cause chorea in a rheumatic subject, or exposure rheumatism in a choreic subject. But soon this double liability ceases. That time of life comes when the reign of chorea is feeble and nearing its end, while rheumatism takes wider expansion, and becomes endowed with many prominent characteristics which have been hitherto absent. But it is needless to pursue the subject further. My main design, indeed, is not to discuss theories, but to call attention to facts which have only now become known to me. Looking at chorea as a whole, I have always sided with those who maintain that its relationship to rheumatism is often exaggerated, and that the manner of the association in certain instances is far more striking than its frequency upon the whole. That is my contention still; but as I now perceive, it is incomplete. A full examination of the question, from the point of view selected in the present paper, compels the further conclusion that chorea and polyarthritis in their pathogenesis are nearly akin, while at the same time, as I venture to think, it suggests the reason why these two affections, although cradling together, should have but a short-lived intimacy, as well as the nature of the agency which soon begins to relax the bond of union and at length altogether dissolves it.—*Archives of Pediatrics, May, 1887.*

